# 6-PPD Quinone Inhibits Phosphatidic Acid Synthesis Associated with an Increase in Intestinal Barrier Permeability in *C. elegans*

**DOI:** 10.3390/toxics14030254

**Published:** 2026-03-12

**Authors:** Jingwei Wu, Qian Bian, Dayong Wang

**Affiliations:** 1 Medical School, Southeast University, Nanjing 210009, China; 2 Jiangsu Provincial Center for Disease Control and Prevention, Nanjing 210009, China

**Keywords:** intestinal barrier, 6-PPDQ, phosphatidic acid, nematodes

## Abstract

6-PPD quinine (6-PPDQ) affects intestinal barrier function; however, its underlying mechanisms remain largely unknown. In the current study, we examined the role of reduction in phosphatidic acid synthesis in mediating the toxicity of 6-PPDQ in affecting intestinal barrier function. In *Caenorhabditis elegans*, 6-PPDQ exposure reduced the phosphatidic acid content, which was accompanied by the decreased expression of *acl-5* and *acl-6* encoding glycerol-3-phosphate acyltransferase. The RNAi of *acl-5* and *acl-6* lowered the phosphatidic acid content, enhanced intestinal permeability, and resulted in the increased accumulation of 6-PPDQ. Meanwhile, *acl-5* and *acl-6* RNAi caused susceptibility to 6-PPDQ toxicity by upregulating the expressions of insulin ligands and receptor genes and downregulating the expressions of *daf-16* and its target genes. Moreover, the RNAi of *acl-5* and *acl-6* elevated the expression of *let-363*, and the RNAi of *let-363* could reduce the expressions of insulin ligand genes and confer resistance to 6-PPDQ toxicity. The double RNAi of *acl-5* and *acl-6* caused more severe enhanced intestinal permeability and 6-PPDQ toxicity. Therefore, 6-PPDQ exposure potentially disrupts phosphatidic acid synthesis to affect intestinal barrier function by downregulating *acl-5* and *acl-6* expressions.

## 1. Introduction

Through different pathways, 6-PPD quinone (6-PPDQ) is generated from 6-PPD [1,2,3]. Besides acting as cause for coho salmon lethality [4], 6-PPDQ exists in different environments, especially in aquatic environments [5,6,7,8]. The range of environmentally relevant concentrations (ERCs) for 6-PPDQ is tens of μg/L or ng/L [4,9,10,11,12]. Exposure to 6-PPDQ causes multiple aspects of toxicity in organisms, including aquatic organisms [13,14,15,16,17,18,19,20]. Exposure to 6-PPDQ also results in organ injury in mammals, such as damage to the liver [21,22,23,24,25]. 6-PPDQ detection from human-related biological samples suggests a potential health risk to human beings [26,27,28,29,30]. After intraperitoneal injection, 6-PPDQ was found to be accumulated in multiple organs of mice [31,32].

*Caenorhabditis elegans* is useful to detect pollutant toxicity [32,33,34,35,36] and to determine its underlying molecular mechanisms [37,38,39,40]. This is largely due to the sensitivity to pollutant exposure in this animal model [41,42,43,44,45]. In nematodes, exposure to 6-PPDQ could cause several aspects of toxicity, including neurotoxicity and reproductive toxicity [46,47,48,49]. Some biochemical metabolisms (such as amino acid and vitamin D3 metabolisms) were disrupted by 6-PPDQ [50,51,52,53]. The organism’s lifespan was further decreased by 6-PPDQ, which was related to dysregulation of insulin/IGF-1 signaling [54], disruption in mitochondrial complexes’ function [55,56,57], and suppression in mitochondrial unfolded protein response (mt UPR) and mitophagy [58,59]. Moreover, 6-PPDQ resulted in disruption of *C. elegans* intestinal barrier function reflected by an enhancement in intestinal permeability, which was associated with the formation of susceptibility to 6-PPDQ toxicity [60]. However, the molecular basis for this observed 6-PPDQ intestinal toxicity is largely unclear.

**Figure 1:**
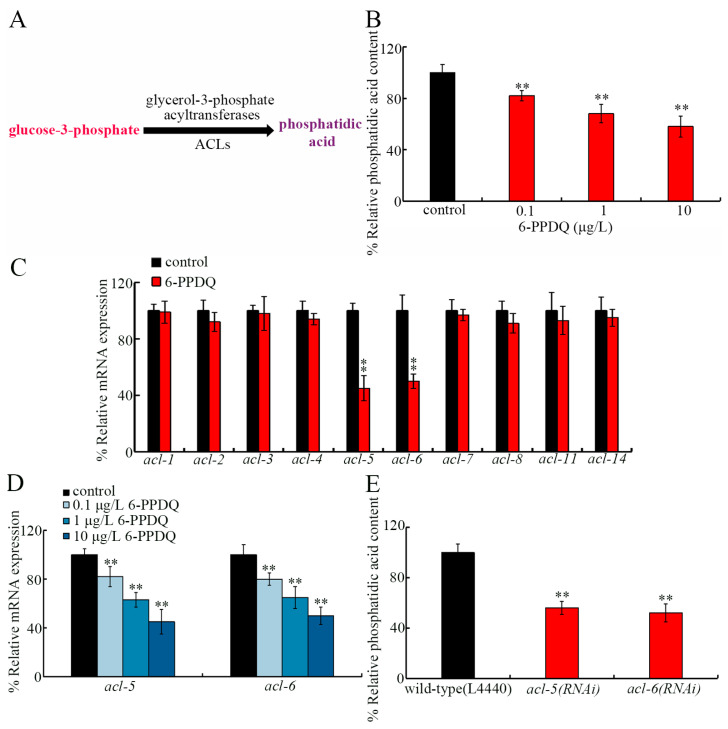
Effect of 6-PPDQ exposure on synthesis of phosphatidic acid. (**A**) A diagram showing control of phosphatidic acid synthesis from glucose-3-phosphate by glucose-3-phosphate acyltransferases in nematodes. (**B**) Effect of 6-PPDQ exposure on phosphatidic acid content in wild-type N2 nematodes. N = 3. ** *p* < 0.01 vs. control. (**C**) Effect of 6-PPDQ (10 μg/L) on expression of intestinal *acl* genes in wild-type N2 nematodes. Thirty intact intestines were isolated for qRT-PCR analysis. N = 3. ** *p* < 0.01 vs. control. (**D**) Effect of 6-PPDQ exposure on expression of intestinal *acl5* and *acl-6* in wild-type N2 nematodes. Thirty intact intestines were isolated for qRT-PCR analysis. N = 3. ** *p* < 0.01 vs. control. (**E**) Effect of RNAi of *acl-5* and *acl-6* on phosphatidic acid content in 6-PPDQ-exposed wild-type N2 nematodes. N = 3. Exposure concentration of 6-PPDQ was 10 μg/L. ** *p* < 0.01 vs. wild-type(L4440). Data are presented as the mean ± standard deviation (SD).

Phosphatidic acid, the simplest diacylglycerophospholipid, can exert diverse biological functions [61], particularly in regulating membrane assembly [62]. Growing evidence has indicated that phosphatidic acid synthesis is essential for maintaining cell membrane integrity [63,64]. We therefore hypothesized that 6-PPDQ might increase intestinal permeability by disrupting phosphatidic acid synthesis. In *C. elegans*, phosphatidic acid is synthesized from glycerol-3-phosphate through acylation reactions catalyzed by acyltransferases encoded by *acl* genes (Figure 1A) [65]. Among *acl* genes, *acl-1–8*, *11*, and *14* can be expressed in the intestine (https://wormbase.org). In mammals, phosphatidic acid is known to bind to target of rapamycin (TOR) [66]. Inhibition in *C. elegans* LET-363/TOR has been shown to extend lifespan [67]. In this study, the effects of 6-PPDQ on phosphatidic acid content and expression of intestinal *acl* genes were first examined. Moreover, the role of candidate *acl* genes in modulating 6-PPDQ toxicity on intestinal barrier function and the underlying mechanism were determined. The findings highlighted the role of inhibition in ACL-5/6 in mediating 6-PPDQ damage to intestinal barrier function and causing susceptibility to 6-PPDQ toxicity by activating the mTOR–insulin signaling axis.

## 2. Materials and Methods

### 2.1. Animal Maintenance

Animal strains (Table S1) were cultured according to standard *C. elegans* protocol [68] on an NGM plate fed with *E. coli* OP50. The used strains are from the Caenorhabditis Genetics Center (CGC). Gravid hermaphrodites were subjected to embryonic isolation using a lysis buffer in order to obtain L1-larvae [69].

### 2.2. Exposure

The 6-PPDQ (Toronto Research Chemicals, Toronto, ON, Canada) exposure concentrations were 0.1–10 μg/L, which correspond to those in actual water environments [4]. 6-PPDQ exposure was from L1-larave and lasted until the third day of adulthood (6.5 days) at 20 °C in darkness [70]. The 6-PPDQ was dissolved in dimethyl sulfoxide (DMSO), and DMSO solution diluted by K buffer in the same way as the 6-PPDQ solutions was used as the control solution. Exposure solutions of 6-PPDQ prepared by diluting the stock solution with K buffer (0.032 M KCl, 0.051 M NaCl) were refreshed daily. Nematodes were exposed to 6-PPDQ solutions with OP50 added as the food source. The exposure volume for each group was 1 mL in 12-well glass plates. Approximately 1000 nematodes were exposed to 6-PPDQ for each group. During the exposure, shaking at 150 rpm was performed for cultured nematodes. Nematodes were randomly assigned to groups for the assessment of different endpoints. During exposure, the 6-PPDQ working solutions were updated daily.

The concentrations of the 6-PPDQ exposure solutions were confirmed by HPLC-MS/MS (PerkinElmer, Waltham, MA, USA). The 6-PPDQ body accumulation was further examined by HPLC-MS/MS, and we provide the detailed experimental procedure in Text S1.

### 2.3. Phosphatidic Acid Content

A phosphatidic acid test kit (Shanghai Chutai Biotechnology Co., Shanghai, China) was used. Nematodes were weighed, homogenized, and centrifuged. Supernatant was measured for absorbance at 450 nm. Experiments were conducted in triplicate.

### 2.4. Endpoints

For the reactive oxygen species (ROS) assay, animals were treated using 1 μM CM-H_2_DCFDA for 2 h with shaking at 200 rpm [71]. Following incubation, ROS fluorescent signals were examined (excitation/emission wavelength: 488/510 nm). For the lipofuscin accumulation assay, fluorescent signals were analyzed under a DAPI filter [72]. Fifty animals were tested. Experiments were conducted in triplicate.

For locomotion assay, head thrashing was examined by observing movement direction along the *X*-axis and changes in the direction of the posterior (*Y*-axis) [71], and body bending was examined by tracking the mid-body bending direction [72]. Brood size refers to the total number of offspring for an individual nematode and was assessed until animals stopped egg-laying [73]. Fifty animals were tested. Experiments were conducted in triplicate.

### 2.5. Intestinal Permeability

Animals were stained using 5% erioglaucine disodium for 3 h [69]. Images were analyzed under bright field. Fifty animals were tested.

### 2.6. Gene Expression

Nematodes were lysed using pre-chilled TRIzol (Sangon Biotech. Co., Ltd., Shanghai, China). cDNA was prepared by M-MuLV reverse transcriptase (Sangon Biotech. Co., Ltd., Shanghai, China). Target gene expressions were assessed by qRT-PCR with *tba-1* serving as reference gene [74]. The primers are available in Table S2.

### 2.7. RNA Interference (RNAi)

L1-larvae were treated on RNAi plates seeded by double-stranded RNA-expressing *E. coli* HT115. Their progeny were exposed to 6-PPDQ. Transgenic strains of VP303 and WM118 are tools for intestinal and muscle gene RNAi. Empty vector/L4440 served as control [74]. Figures S1 and S2 show the RNAi efficiency.

### 2.8. Data Analysis

One-way or two-way ANOVA (for multi-factor comparison) followed by the Tukey post hoc test was used to evaluate differences between different groups. A *p*-value of <0.01 (**) was deemed statistically significant.

## 3. Results

### 3.1. 6-PPDQ Inhibited Synthesis of Phosphatidic Acid

Phosphatidic acid content was decreased by 6-PPDQ (Figure 1B). Among intestinal *acl* genes, expressions of *acl-1–4*, *acl-6*, *acl-8*, *acl-11*, and *acl-14* were not changed by 6-PPDQ; however, *acl-5* and *acl-6* expressions were reduced by 6-PPDQ (Figure 1C). After 6-PPDQ exposure, the decrease in *acl-5* and *acl-6* expressions were concentration dependent (Figure 1D). Moreover, phosphatidic acid content was inhibited by *acl-5* and *acl-6* RNAi (Figure 1E).

### 3.2. acl-5 and acl-6 RNAi Induced Susceptibility to 6-PPDQ Toxicity

**Figure 2:**
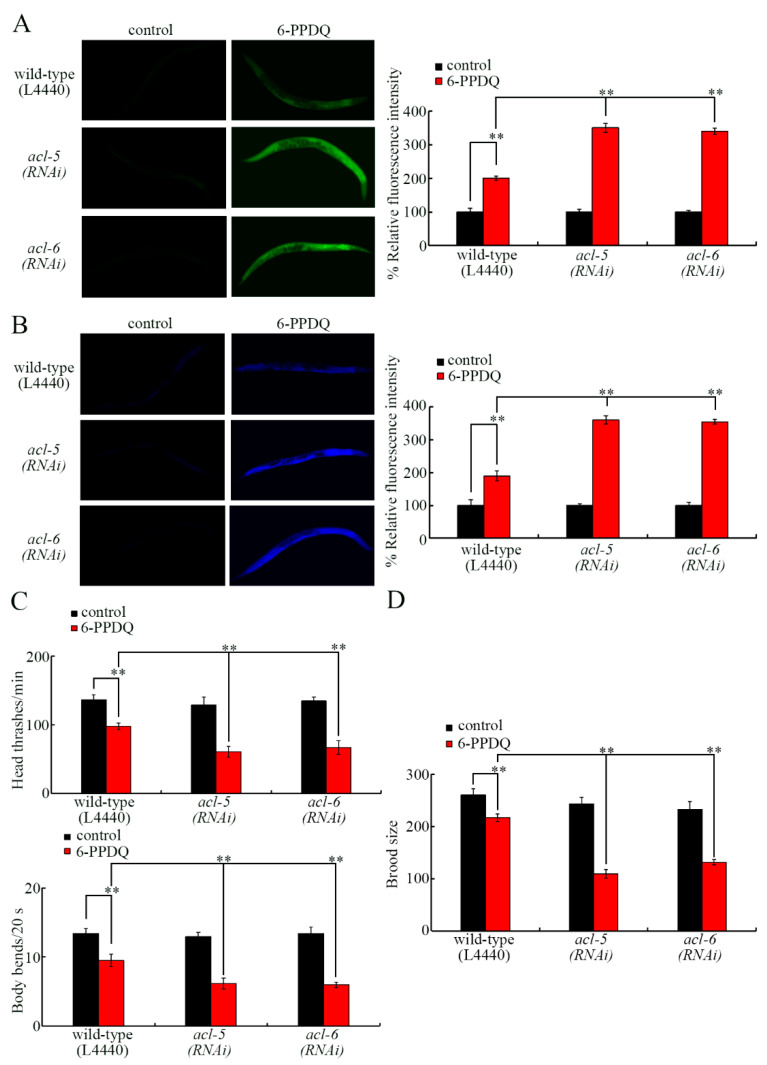
Effect of RNAi of *acl-5* and *acl-6* on 6-PPDQ toxicity in causing ROS generation (**A**), inducing intestinal lipofuscin accumulation (**B**), decreasing locomotion (**C**), and reducing brood size (**D**) in wild-type N2 nematodes. N = 50. Exposure concentration of 6-PPDQ was 10 μg/L. ** *p* < 0.01. Data are presented as the mean ± standard deviation (SD).

Using lipofuscin accumulation and ROS generation as intestinal-toxicity-related endpoints, the RNAi of *acl-5* and *acl-6* caused more severe 6-PPDQ intestinal toxicity in inducing lipofuscin accumulation and ROS generation compared with those in wild-type N2 nematodes (Figure 2A,B). Using locomotion as a neurotoxicity-related endpoint and brood size as a reproductive-toxicity-related endpoint, more severe 6-PPDQ neurotoxicity in inhibiting locomotion and reproductive toxicity in reducing brood size were caused by *acl-5* and *acl-6* RNAi compared with those in wild-type N2 nematodes (Figure 2C,D).

### 3.3. Tissue-Specific Activity of ACL-5 and ACL-6 to Control 6-PPDQ Toxicity

**Figure 3:**
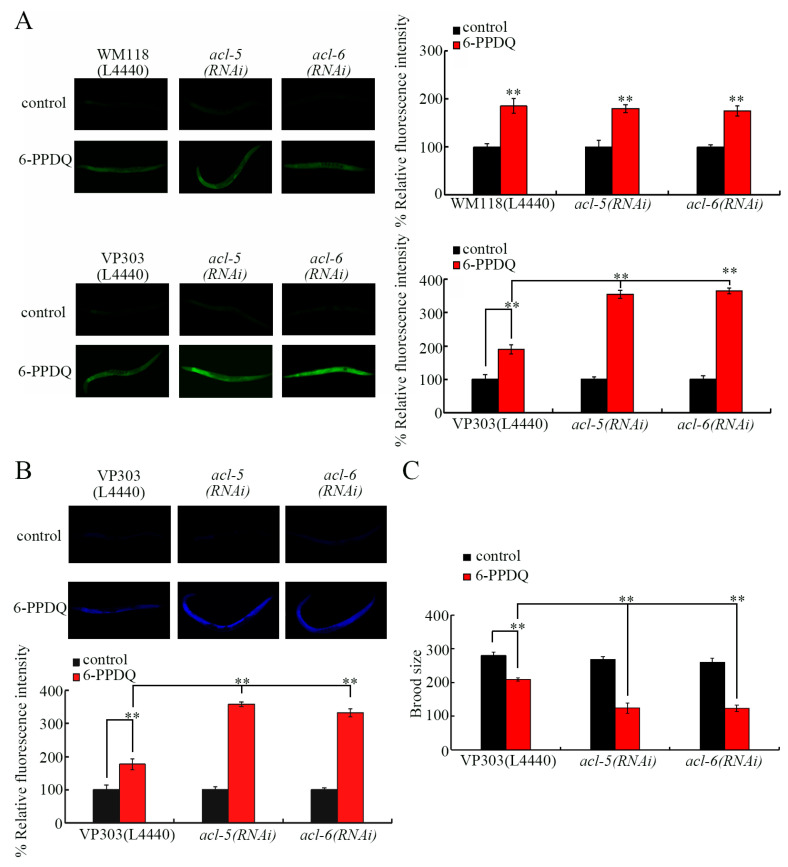
Tissue-specific activity of ACL-5 and ACL-6 in regulating 6-PPDQ toxicity. (**A**) Tissue-specific activity of ACL-5 and ACL-6 in regulating 6-PPDQ toxicity in causing intestinal ROS generation. N = 50. (**B**) Intestine-specific activity of ACL-5 and ACL-6 in regulating 6-PPDQ toxicity in causing intestinal lipofuscin accumulation. N = 50. (**C**) Intestine-specific activity of ACL-5 and ACL-6 in regulating 6-PPDQ toxicity in reducing brood size. Exposure concentration of 6-PPDQ was 10 μg/L. N = 50. ** *p* < 0.01. Data are presented as the mean ± standard deviation (SD).

We next focused on ACL-5 and ACL-6 to examine their tissue-specific activity in controlling 6-PPDQ toxicity. *C. elegans* ACL-5 and ACL-6 are expressed in the muscle and intestine (https://wormbase.org). Nevertheless, using intestinal ROS generation as an endpoint, induction of intestinal ROS generation by 6-PPDQ was not changed by the muscle RNAi of *acl-5*, and *acl-6* but could be increased by the intestinal RNAi of *acl-5* and *acl-6* (Figure 3A). Similarly, 6-PPDQ-caused lipofuscin accumulation was strengthened by intestinal *acl-5* and *acl-6* RNAi (Figure 3B). Additionally, 6-PPDQ-caused reproductive reduction was increased by intestinal *acl-5* and *acl-6* RNAi (Figure 3C).

### 3.4. ACL-5 and ACL-6 Modulated Intestinal Permeability

**Figure 4:**
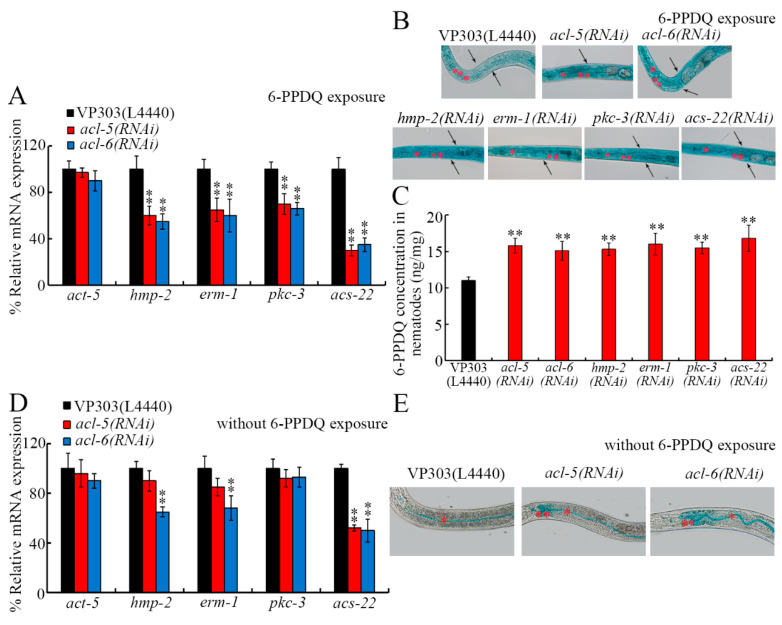
Effect of intestinal RNAi of *acl-5* and *acl-6* on intestinal permeability under 6-PPDQ exposure or without 6-PPDQ exposure condition. (**A**) Effect of intestinal RNAi of *acl-5* and *acl-6* on expression of genes governing functional state of intestinal barrier in 6-PPDQ-exposed VP303 nematodes. Exposure concentration of 6-PPDQ was 10 μg/L. N = 3. ** *p* < 0.01 vs. VP303(L4440). (**B**) Effect of intestinal RNAi of *acl-5*, *acl-6*, *hmp-2*, *erm-1*, *acs-22*, and *pkc-3* on intestinal permeability in 6-PPDQ-exposed VP303 nematodes. Exposure concentration of 6-PPDQ was 10 μg/L. Single asterisks and double asterisks indicate intestinal lumen and intestinal cells, respectively. Arrowheads indicate the body cavity. N = 50. (**C**) Effect of intestinal RNAi of *acl-5*, *acl-6*, *hmp-2*, *erm-1*, *acs-22*, and *pkc-3* on 6-PPDQ accumulation in body of VP303 nematodes after exposure to 10 μg/L 6-PPDQ. N = 3. ** *p* < 0.01 vs. VP303(L4440). (**D**) Effect of intestinal RNAi of *acl-5* and *acl-6* on expression of genes governing functional state of intestinal barrier in VP303 nematodes under the condition without 6-PPDQ exposure N = 3. ** *p* < 0.01 vs. VP303(L4440). (**E**) Effect of intestinal RNAi of *acl-5* and *acl-6* on intestinal permeability in VP303 nematodes under the condition without 6-PPDQ exposure. Single asterisks and double asterisks indicate intestinal lumen and intestinal cells, respectively. N = 50. Data are presented as the mean ± standard deviation (SD).

Some *C. elegans* proteins are shown to regulate intestinal permeability [72]. Considering the important association between phosphatidic acid metabolism and cell membrane integrity [63], we next examined the role of ACL-5 and ACL-6 in modulating the functional state of the intestinal barrier. In 6-PPDQ-exposed nematodes, although *act-5* expression was not changed by *acl-5* and *acl-6* RNAi, expressions of *pkc-3*, *erm-1*, *hmp-2*, and *acs-22* were decreased by *acl-5* and *acl-6* RNAi (Figure 4A). The intestinal permeability could be strengthened by 6-PPDQ [60]. The intestinal permeability of 6-PPDQ-exposed nematodes was enhanced not only by *acl-5* and *acl-6* RNAi but also by *pkc-3*, *hmp-2*, *erm-1*, and *acs-22* RNAi (Figure 4B). Moreover, 6-PPDQ accumulation was increased by *acl-5*, *acl-6*, *pkc-3*, *hmp-2*, *erm-1*, and *acs-22* RNAi (Figure 4C).

Under normal conditions, although *act-5*, *hmp-2*, *erm-1*, and *pkc-3* expressions were not affected by intestinal *acl-5* RNAi, *acs-22* expression was decreased by the intestinal RNAi of *acl-5* (Figure 4D). Moreover, under normal conditions, expression of *hmp-2*, *erm-1*, and *acs-22* could be decreased by *acl-6* RNAi (Figure 4D). Under normal conditions, intestinal *acl-5* and *acl-6* RNAi could already even cause blue dye translocation to intestinal cells (Figure 4E).

### 3.5. Intestinal RNAi of acl-5 and acl-6 Affected Expression of Genes in Insulin Signaling Pathway

**Figure 5:**
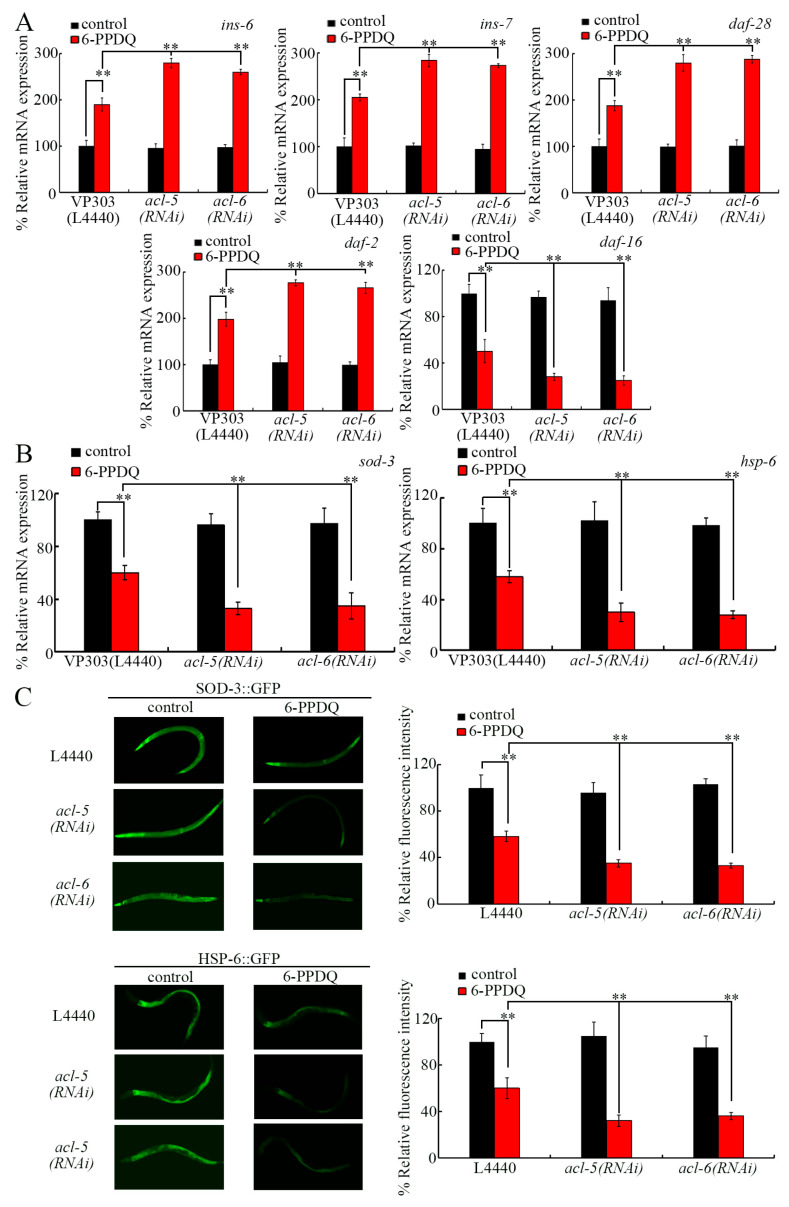
Effect of intestinal RNAi of *acl-5* and *acl-6* on expression of genes in insulin signaling pathways after 6-PPDQ exposure. (**A**) Effect of intestinal RNAi of *acl-5* and *acl-6* on expressions of *ins-6*, *ins-7*, *daf-28*, *daf-2*, and *daf-16* in VP303 nematodes after 6-PPDQ exposure. N = 3. (**B**) Effect of intestinal RNAi of *acl-5* and *acl-6* on expressions of *sod-3* and *hsp-6* in VP303 nematodes after 6-PPDQ exposure. N = 3. (**C**) Effect of RNAi of *acl-5* and *acl-6* on expressions of SOD-3::GFP and HSP-6::GFP in CF1553 and SJ410 nematodes after 6-PPDQ exposure. N = 50. Exposure concentration of 6-PPDQ was 10 μg/L. ** *p* < 0.01. Data are presented as the mean ± standard deviation (SD).

Insulin signaling functions in the *C. elegans* intestine to control pollutant toxicity [72]. Insulin ligands modulated the effect of 6-PPDQ toxicity on longevity by activating receptor DAF-2 and inhibiting DAF-16 and targets (SOD-3 and HSP-6) [75]. Among previously identified insulin ligand genes dysregulated by 6-PPDQ [75], the 6-PPDQ-caused increase in expression of *ins-6*, *ins-7*, and *daf-28* was strengthened by intestinal *acl-5* and *acl-6* RNAi (Figure 5A). Additionally, the 6-PPDQ-caused increase in *daf-2* expression and decrease in *daf-16* expression were enhanced by intestinal *acl-5* and *acl-6* RNAi (Figure 5A). Moreover, the decrease in *sod-3*, *hsp-6*, SOD-3::GFP, and HSP-6::GFP expressions induced by 6-PPDQ was enhanced by intestinal *acl-5* and *acl-6* RNAi (Figure 5B,C).

### 3.6. Effect of Intestinal hmp-2, pkc-3, erm-1, acs-22, daf-28, ins-7, ins-6, daf-2, daf-16, sod-3, and hsp-6 RNAi on 6-PPDQ Toxicity

Using several endpoints, 6-PPDQ toxicity was increased by intestinal *erm-1*, *hmp-2*, *pkc-3*, *acs-22*, *daf-16*, *sod-3*, and *hsp-6* RNAi (Figure S3A–C). Different from this, the 6-PPDQ-caused toxicity was inhibited by intestinal *daf-28*, *ins-7*, *ins-6*, and *daf-2* RNAi (Figure S3A–C).

### 3.7. ACL-5 and ACL-6 Modulated Insulin Signals by Inhibiting LET-363

**Figure 6:**
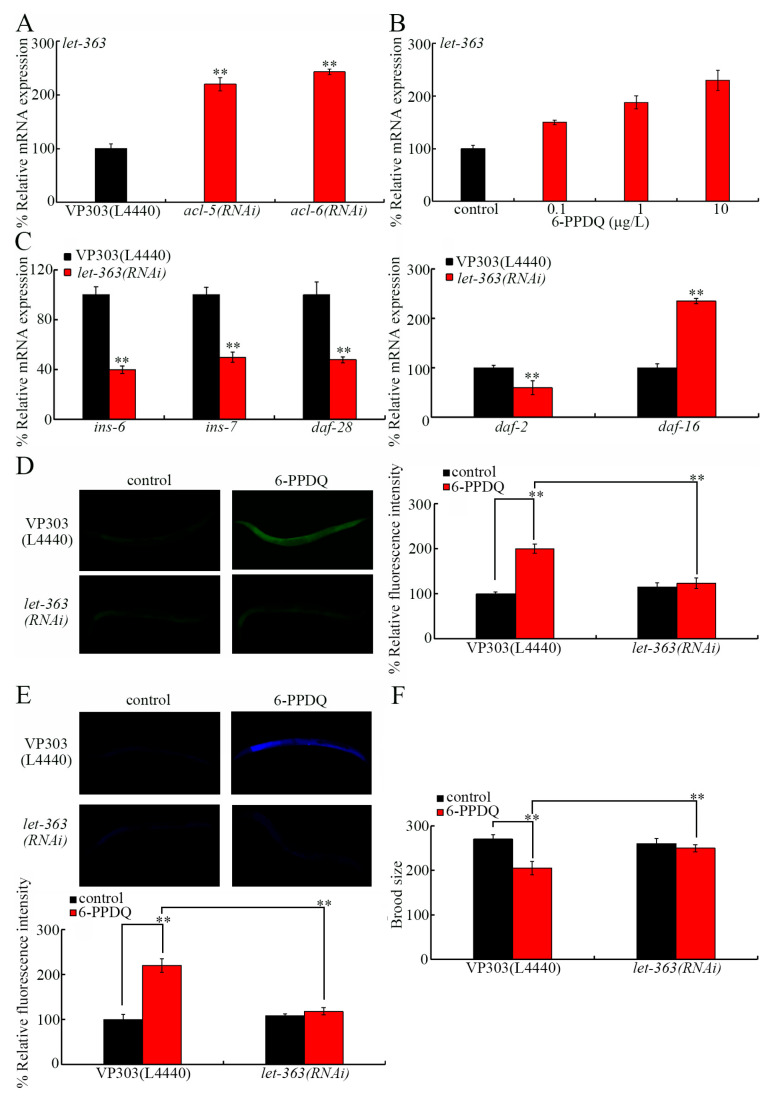
Role of LET-363 in controlling 6-PPDQ toxicity by activating insulin signals. (**A**) Effect of intestinal RNAi of *acl-5* and *acl-6* on expression of *let-363* in 6-PPDQ-exposed VP303 nematodes. Exposure concentration of 6-PPDQ was 10 μg/L. N = 3. ** *p* < 0.01 vs. VP303(L44440). (**B**) Effect of 6-PPDQ exposure on intestinal *let-363* expression in wild-type N2 nematodes. Thirty intact intestines were isolated for qRT-PCR analysis. N = 3. ** *p* < 0.01 vs. control. (**C**) Effect of intestinal RNAi of *let-363* on expression of *ins-6*, *ins-7*, *daf-28*, *daf-2*, and *daf-16* in 6-PPDQ-exposed VP303 nematodes. Exposure concentration of 6-PPDQ was 10 μg/L. N = 3. ** *p* < 0.01 vs. VP303(L4440). (**D**) Effect of intestinal RNAi of *let-363* on 6-PPDQ toxicity in causing intestinal ROS generation in VP303 nematodes. Exposure concentration of 6-PPDQ was 10 μg/L. N = 50. ** *p* < 0.01. (**E**) Effect of intestinal RNAi of *let-363* on 6-PPDQ toxicity in inducing intestinal lipofuscin accumulation in VP303 nematodes. Exposure concentration of 6-PPDQ was 10 μg/L. N = 50. ** *p* < 0.01. (**F**) Effect of intestinal RNAi of *let-363* on 6-PPDQ toxicity in reducing brood size in VP303 nematodes. Exposure concentration of 6-PPDQ was 10 μg/L. N = 50. ** *p* < 0.01. Data are presented as the mean ± standard deviation (SD).

In mammals, phosphatidic acid can by sensed by and bind to TOR [66]. LET-363 is *C. elegans* TOR [76]. *let-363* expression was increased by intestinal *acl-5* and *acl-6* RNAi (Figure 6A). 6-PPDQ increased intestinal *let-363* expression (Figure 6B). The intestinal RNAi of *let-363* decreased *daf-28*, *ins-7*, *ins-6*, and *daf-2* expressions and increased *daf-16* expression (Figure 6C). Besides this, after 6-PPDQ exposure, *let-363* RNAi further increased *sod-3* and *hsp-6* expressions (Figure S4). 6-PPDQ-caused toxicity was inhibited by intestinal *let-363* RNAi (Figure 6D–F).

### 3.8. Effect of Double RNAi of acl-5 and acl-6 on Intestinal Permeability and 6-PPDQ Toxicity Induction

**Figure 7:**
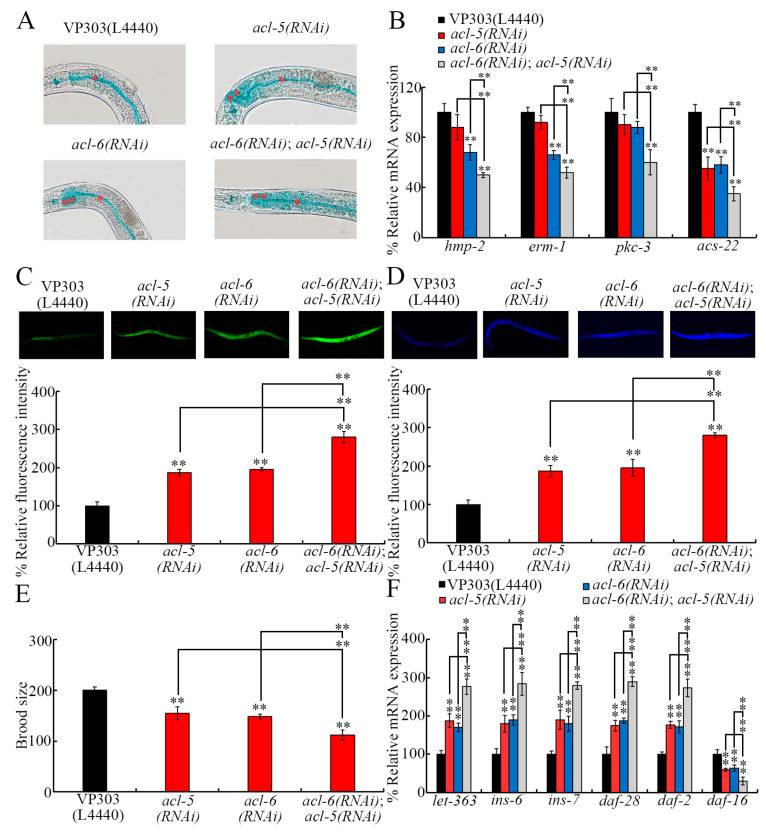
Effect of double RNAi of *acl-5* and *acl-6* on intestinal permeability and 6-PPDQ toxicity induction. (**A**) Effect of double RNAi of *acl-5* and *acl-6* on intestinal permeability in VP303 nematodes. Single asterisks and double asterisks indicate intestinal lumen and intestinal cells. N = 50. (**B**) Effect of double RNAi of *acl-5* and *acl-6* on expressions of *hmp-2*, *erm-1*, *pkc-3*, and *acs-22* in VP303 nematodes. N = 3. ** *p* < 0.01 vs. VP303(L4440) (if not specially indicated). (**C**) Interaction between *acl-5* and *acl-6* in regulating 6-PPDQ toxicity in inducing intestinal ROS generation in VP303 nematodes. Exposure concentration of 6-PPDQ was 10 μg/L. N = 50. ** *p* < 0.01 vs. VP303(L4440) (if not specially indicated). (**D**) Interaction between *acl-5* and *acl-6* in regulating 6-PPDQ toxicity in inducing intestinal lipofuscin accumulation in VP303 nematodes. Exposure concentration of 6-PPDQ was 10 μg/L. N = 50. ** *p* < 0.01 vs. VP303(L4440) (if not specially indicated). (**E**) Interaction between *acl-5* and *acl-6* in regulating 6-PPDQ toxicity in reducing brood size in VP303 nematodes. Exposure concentration of 6-PPDQ was 10 μg/L. N = 50. ** *p* < 0.01 vs. VP303(L4440) (if not specially indicated). (**F**) Effect of double RNAi of *acl-5* and *acl-6* on expressions of *let-363*, *ins-6*, *ins-7*, *daf-28*, *daf-2*, and *daf-16* in 6-PPDQ-exposed VP303 nematodes. Exposure concentration of 6-PPDQ was 10 μg/L. N = 3. ** *p* < 0.01 vs. VP303(L4440) (if not specially indicated). Data are presented as the mean ± standard deviation (SD).

After double RNAi of *acl-5* and *acl-6*, more severe intestinal permeability was detected in *acl-6*(*RNAi*);*acl-5*(*RNAi*) than in *acl-5*(*RNAi*) and *acl-6*(*RNAi*) (Figure 7A). Accompanying this, more severe decrease in *hmp-2*, *erm-1*, and *acs-22* expressions were found in *acl-6*(*RNAi*);*acl-5*(*RNAi*) than in *acl-5*(*RNAi*) and/or *acl-6*(*RNAi*) (Figure 7B). Compared with no expressional change of *pkc-3* in *acl-5*(*RNAi*) and/or *acl-6*(*RNAi*), *pkc-3* expression was reduced in *acl-6*(*RNAi*);*acl-5*(*RNAi*) (Figure 7B).

Moreover, more severe 6-PPDQ toxicity was found in *acl-6*(*RNAi*);*acl-5*(*RNAi*) than in *acl-5*(*RNAi*) and/or *acl-6*(*RNAi*) (Figure 7C–E). Meanwhile, more severe increases in *let-363*, *daf-28*, *ins-6*, *ins-7*, and *daf-2* expressions and decrease in *daf-16* expression were also detected in *acl-6*(*RNAi*);*acl-5*(*RNAi*) than in *acl-5*(*RNAi*) and/or *acl-6*(*RNAi*) (Figure 7F).

## 4. Discussion

The intestine is the largest organ in nematodes. After 6-PPDQ exposure, *C. elegans* intestinal permeability could be enhanced [60], which suggests disrupted intestinal function. Considering the role of phosphatidic acid during cell membrane organization [62], we determined the effect of 6-PPDQ exposure on phosphatidic acid synthesis and its association with 6-PPDQ toxicity, especially the damage to intestinal function. We here observed a reduction in phosphatidic acid content, which was due to inhibition of its synthesis. Accompanying the reduction in phosphatidic acid content (Figure 1B), *acl-5* and *acl-6* expressions were decreased by 6-PPDQ (Figure 1C), and phosphatidic acid content could be reduced by *acl-5* and *acl-6* RNAi (Figure 1E). ACL-5 is the homolog of mammal endoplasmic reticulum (ER) GPAT4, and ACL-6 is the homolog of mammal mitochondrial GPAT1 and GPAT2 [77]. ACLs are predicted to catalyze the process generating phosphatidic acid, the precursor of membrane phospholipids [77]. Among intestinal *acl* genes encoding glycerol-3-phosphate acyltransferases, only the expressions of *acl-5* and *acl-6* were decreased by 6-PPDQ (Figure 1C). That is, the expression of not all intestinal *acl* genes had enough sensitivity to show a response to 6-PPDQ. Among these intestinal *acl* genes, our data suggested that expression of *acl-5* and *acl-6* may be highly sensitive after pollutant exposure. The reduction in phosphatidic acid content and decrease in *acl-5* and *acl-6* expression by 6-PPDQ suggested that 6-PPDQ at ERCs could reduce phosphatidic acid content by inhibiting certain glycerol-3-phosphate acyltransferases.

We further provide several lines of evidence to demonstrate that the decrease in *acl-5* and *acl-6* expressions mediated the induction of 6-PPDQ toxicity. That is, accompanied with the decrease in *acl-5* and *acl-6* expressions (Figure 1D), *acl-5* and *acl-6* RNAi caused susceptibility to 6-PPDQ toxicity. *acl-5*(*RNAi*) and *acl-6*(*RNAi*) showed susceptibility to 6-PPDQ toxicity at different aspects. Using intestinal endpoints, *acl-5* and *acl-6* RNAi induced susceptibility to 6-PPDQ intestinal toxicity (Figure 2A,B). Using locomotion as the endpoint, *acl-5* and *acl-6* RNAi caused susceptibility to 6-PPDQ neuronal toxicity (Figure 2C). Using brood size as the endpoint, *acl-5* and *acl-6* RNAi also induced susceptibility to 6-PPDQ reproductive toxicity (Figure 2D). Further examination of the effect of *acl-5* and *acl-6* overexpression is suggested in the future to further confirm these observations.

Moreover, ACL-5 and ACL-6 functioned in the intestine to control 6-PPDQ toxicity. The observations suggested that susceptibility of *acl-5*(*RNAi*) and *acl-6*(*RNAi*) to 6-PPDQ intestinal toxicity might be the direct effect of the RNAi of *acl-5* and *acl-6*. In contrast, the detected susceptibility of *acl-5*(*RNAi*) and *acl-6*(*RNAi*) to 6-PPDQ neuronal toxicity and reproductive toxicity might be the indirect effect of the RNAi of *acl-5* and *acl-6*.

For the mechanism of reduction in *acl-5* and *acl-6* expressions in mediating the 6-PPDQ toxicity on intestinal barrier function, we first found that more severe enhancement in intestinal permeability was caused by the RNAi of *acl-5* and *acl-6* after 6-PPDQ exposure (Figure 4B), which was accompanied by inhibited *pkc-3*, *erm-1*, *hmp-2*, and *acs-22* expression (Figure 4A). The *hmp-2* encodes beta-catenin [78]; *erm-1* encodes ezrin–radixin–moesin [79]; *pkc-3* encodes atypical protein kinase C [80]; and *acs-22* encodes fatty acid acyl-CoA synthetase [81]. Mutation or RNAi of these genes causes increased intestinal permeability [82,83]. Meanwhile, we observed an increase in 6-PPDQ accumulation by the RNAi of *acl-5*, *acl-6*, *pkc-3*, *erm-1*, *hmp-2*, and *acs-22* (Figure 4C). The intestinal RNAi of *acl-5* and *acl-6* could further cause enhanced intestinal permeability (Figure 4E). For the molecular basis of this enhanced intestinal permeability, intestinal *acl-5* RNAi decreased *acs-22* expression, and intestinal *acl-6* RNAi decreased *erm-1*, *hmp-2*, and *acs-22* expression (Figure 4D). Therefore, the observed more severe enhancement in intestinal permeability in 6-PPDQ-exposed *acl-5/6*(*RNAi*) was not only associated with ACL-5 and ACL-6 functions but also closely related to the role of PKC-3, ERM-1, HMP-2, and ACS-22. Nevertheless, besides the decrease in their expressions detected in *acl-5*(*RNAi*) and/or *acl-6*(*RNAi*) under normal conditions, *pkc-3* expression was also decreased in 6-PPDQ-exposed *acl-5*(*RNAi*) and/or *acl-6*(*RNAi*) (Figure 4A). These may be because the intestinal RNAi of *acl-5* and *acl-6* induced susceptibility to 6-PPDQ damage (Figure 3), which further induced the decrease in *pkc-3* expression. Furthermore, the intestinal RNAi of *acl-5* and *acl-6* and their downstream target genes induced susceptibility to 6-PPDQ damage (Figure 3 and Figure S3). After pollutant exposure, intestinal oxidative damage contributed to enhanced intestinal permeability [72]. Additionally, after 6-PPDQ exposure, these targeted genes’ expression was negatively correlated with intestinal oxidative stress [84]. That is, the induced oxidative stress might act together with the decrease in *acl-5/6* and their target gene expressions to lead to the induction of enhanced intestinal permeability and 6-PPDQ accumulation.

For the effects of RNAi of *acl-5* and *acl-6* on the intestine, we further found that the intestinal RNAi of *acl-5* and *acl-6* induced susceptibility to 6-PPDQ toxicity by affecting the insulin signaling pathway. In the insulin signaling pathway, activation of insulin ligands and receptor DAF-2 and inhibition in DAF-16 and its targets mediated 6-PPDQ toxicity [75]. Moreover, the increased expressions of these ligands and receptor genes and the decrease in expressions of *daf-16* and its two target genes were strengthened by *acl-5/6* RNAi (Figure 5A–C). Meanwhile, 6-PPDQ-caused intestinal toxicity and reproductive toxicity were inhibited by the intestinal RNAi of these ligands and receptor genes and enhanced by the RNAi of *daf-16* and its two target genes (Figure S3A–C). Therefore, the intestinal RNAi of *acl-5* and *acl-6* induced susceptibility to 6-PPDQ damage by activating insulin ligands and DAF-2 and inhibiting DAF-16 and its targets.

In cells, TOR acted as the sensor of phosphatidic acid [66]. LET-363 is the *C. elegans* TOR [85]. Moreover, we identified that the intestinal RNAi of *acl-5* and *acl-6* induced susceptibility to 6-PPDQ toxicity through the LET-363/TOR–insulin signaling axis. *let-363* expression was activated by 6-PPDQ (Figure 6B), and its expression was accelerated by *acl-5* and *acl-6* RNAi (Figure 6A). Meanwhile, 6-PPDQ intestinal toxicity and reproductive toxicity were suppressed by *let-363* RNAi (Figure 6D–F). More importantly, insulin ligand genes and *daf-2* expressions were decreased by *let-363* RNAi (Figure 6C), and *daf-16* and target gene expressions were increased by *let-363* RNAi (Figure 6C and Figure S4). Therefore, the 6-PPDQ-caused reduction in phosphatidic acid induced the increase in intestinal *let-363* expression, which in turn activated certain intestinal insulin ligands and their receptors to mediate the induction of 6-PPDQ toxicity.

Furthermore, more severely enhanced intestinal permeability and decreased expression of related genes were found in *acl-6*(*RNAi*);*acl-5*(*RNAi*) than in *acl-5*(*RNAi*) and *acl-6*(*RNAi*) (Figure 7A,B). This provides an important mechanism to amplify 6-PPDQ damage to intestinal barrier function. The alteration in *pkc-3*, *erm-1*, *hmp-2*, and *acs-22* expressions reflects the corresponding molecular basis for this amplification mechanism. Following this change, more severe 6-PPDQ toxicity was further observed in *acl-6*(*RNAi*);*acl-5*(*RNAi*) (Figure 7C–E). The more severe increase in expressions of *let-363* and insulin ligand and receptor genes and decrease in *daf-16* expression provide a molecular basis for the induction of this more severe 6-PPDQ toxicity to a certain degree.

## 5. Conclusions

In conclusion, phosphatidic acid content was reduced by 6-PPDQ. This reduction in phosphatidic acid content in 6-PPDQ-exposed nematodes was due to a decrease in the expressions of *acl-5* and *acl-6* governing phosphatidic acid synthesis. ACL-5 and ACL-6 functioned in the intestine to control 6-PPDQ toxicity. On the one hand, intestinal *acl-5* and *acl-6* RNAi disrupted intestinal barrier function reflected by enhanced intestinal permeability and a decrease in intestinal *erm-1*, *hmp-2*, and/or *acs-22* expression. On the other hand, *acl-5* and *acl-6* RNAi caused susceptibility to 6-PPDQ by activating the intestinal TOR–insulin signaling axis. The double RNAi of *acl-5* and *acl-6* resulted in a more severe defect in intestinal permeability and 6-PPDQ toxicity. Our results provide an important molecular basis for 6-PPDQ in causing damage to intestinal barrier function. Additionally, the 6-PPDQ exposure risk in disrupting the phosphatidic acid metabolism of organisms is suggested.

## Data Availability

The original data presented in this study are included in the article/Supplementary Materials; further inquiries can be directed to the corresponding author.

## Supplementary Materials

The following supporting information can be downloaded at https://www.mdpi.com/article/10.3390/toxics14030254/s1: Text S1: Extraction and instrumental analysis of 6-PPDQ. Figure S1: RNAi efficiency of *acl-5* and *acl-6*. ** *p* < 0.01 vs. wild-type(L4440), WM118(L4440), or VP303(L4440). Figure S2: RNAi efficiency of *hmp-2*, *erm-1*, *pkc-3*, *acs-22*, *ins-6*, *ins-7*, *daf-28*, *daf-2*, *daf-16*, *sod-3*, *hsp-6*, and *let-363*. ** *p* < 0.01 vs. VP303(L4440). Figure S3: Effect of intestinal RNAi of *hmp-2*, *erm-1*, *pkc-3*, *acs-22*, *ins-6*, *ins-7*, *daf-28*, *daf-2*, *daf-16*, *sod-3*, and *hsp-6* on 6-PPDQ toxicity in causing intestinal ROS generation (**A**), in inducing intestinal lipofuscin accumulation (**B**), and in reducing brood size (**C**). Exposure concentration of 6-PPDQ was 10 μg/L. ** *p* < 0.01. Figure S4: Effect of RNAi of *let-363* on expression of SOD-3 and HSP-6 in 6-PPDQ-exposed nematodes. (**A**) Effect of RNAi of *let-363* on expression of *sod-3* and *hsp-6* in 6-PPDQ-exposed nematodes. (**B**) Effect of RNAi of *let-363* on expression of SOD-3::GFP in 6-PPDQ-exposed nematodes. (**C**) Effect of RNAi of *let-363* on expression of HSP-6::GFP in 6-PPDQ-exposed nematodes. Exposure concentration of 6-PPDQ was 10 μg/L. ** *p* < 0.01. Table S1: Information for *C. elegans* strains. Table S2: Primer information for qRT-PCR.
