# Supplementary material for: 6-PPD Quinone Inhibits Phosphatidic Acid Synthesis Associated with an Increase in Intestinal Barrier Permeability in C. elegans
Source: Toxics. 2026 Mar 12;14(3):254. doi: 10.3390/toxics14030254 (PMC13030096; doi:10.3390/toxics14030254)
Supplement: Supplementary file 1 [file toxics-14-00254-s001.zip › toxics-4137674-supplementary.pdf]

**Supporting Information:**

**Text S1. Extraction and instrumental analysis of 6-PPDQ**

Nematodes samples were homogenized adding 1 mL acetonitrile for 15 min and repeated three times. After that, samples were centrifuged for 10 min at 12000 g to collect the supernatant. The combined supernatant was transferred to clean-up tubes containing 150 mg $MgSO_4$ and 50 mg C18 (Waters, USA). Tubes were vortexed for two min, and the supernatant was collected after centrifugation at 5000 g for 5 min. Extracts were concentrated by nitrogen gas, and dissolved in 200 μL acetonitrile. Prior to instrumental analysis, solution was filtered with a 0.22 μm filter membrane and stored in polypropylene vials.

6-PPDQ concentrations were performed through HPLC-MS/MS system of Ultimate 3000/Q Exactive (UHPLC-Q-Obitrap MS, Thermo Scientific, USA). Initially, samples were separated using a BEH-C18 column (2.1 mm×100 mm, 1.7 μm film thickness). The used mobile phases were (A) 0.1% formic acid in water and (B) methanol, and the flow rate was 0.4 mL/min. 6-PPDQ level was calculated in positive electrospray ionization (ESI+) mode according to the standard curve. The ion pair of the quantitative transition of 6-PPDQ was m/z 299.17 and m/z 241.09. The ion pair of confirmation transitions of 6-PPDQ was m/z 299.17 and m/z 215.07. The collision energies of the quantitative transition and confirmation transition are both 30 eV. The experiment was done in triplicate each group.

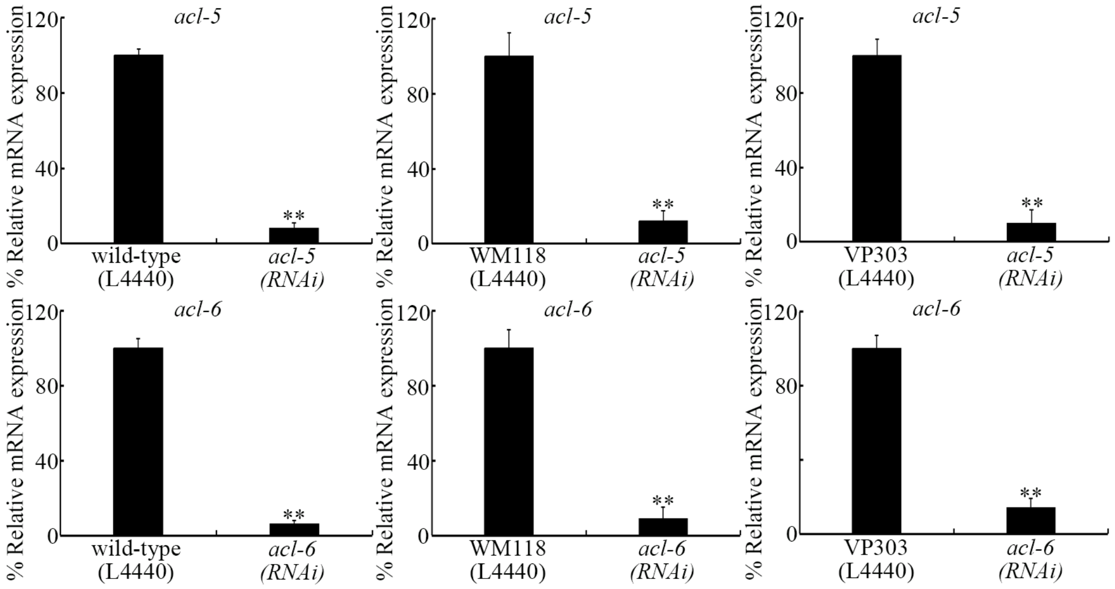


**Figure S1.** RNAi efficiency of *acl-5* and *acl-6*. **$P < 0.01$ *vs* wild-type(L4440), WM118(L4440), or VP303(L4440).

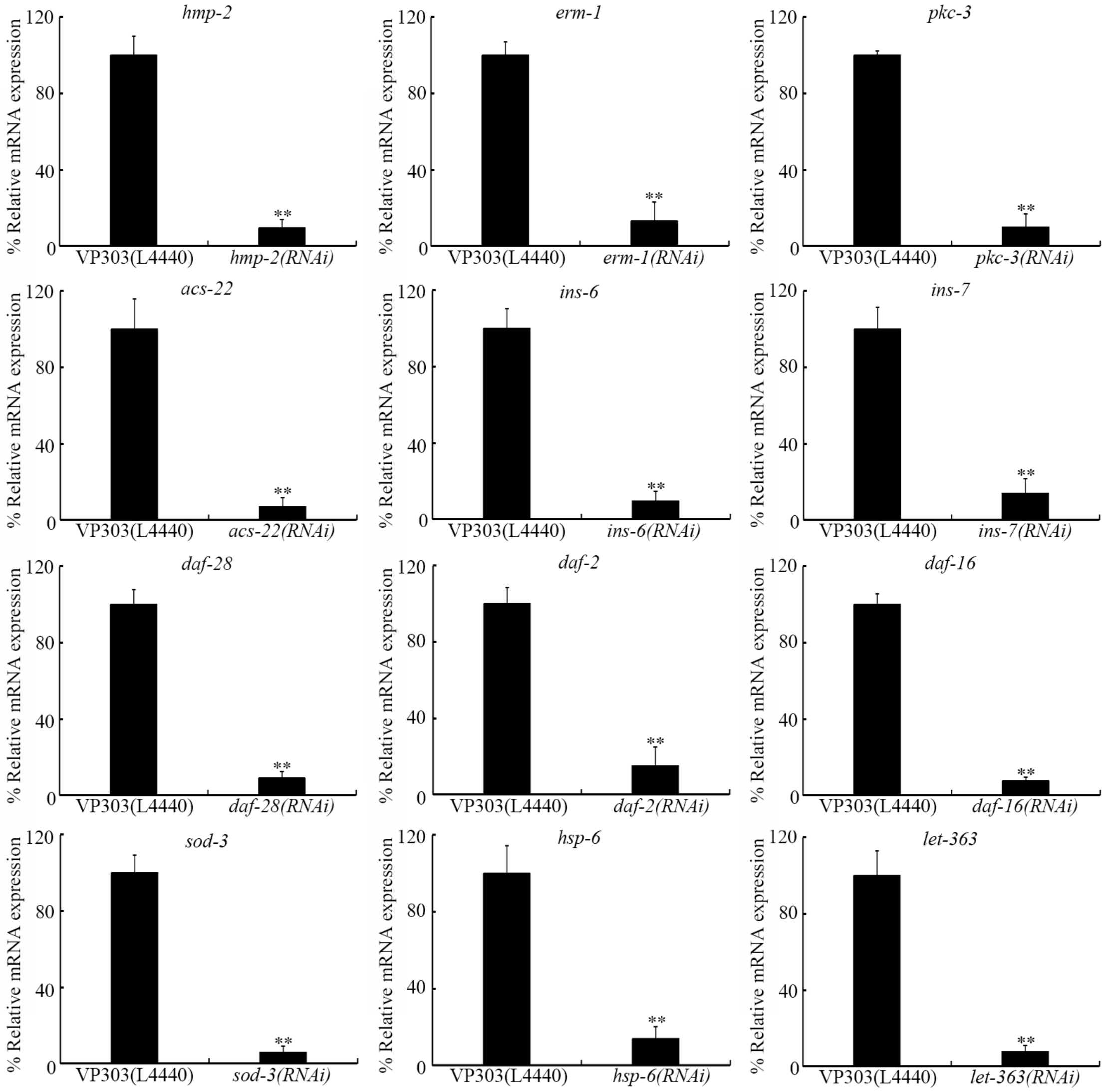


**Figure S2.** RNAi efficiency of *hmp-2, erm-1, pkc-3, acs-22, ins-6, ins-7, daf-28, daf-2, daf-16, sod-3, hsp-6*, and *let-363*. $^{**}P < 0.01$ *vs* VP303(L4440).

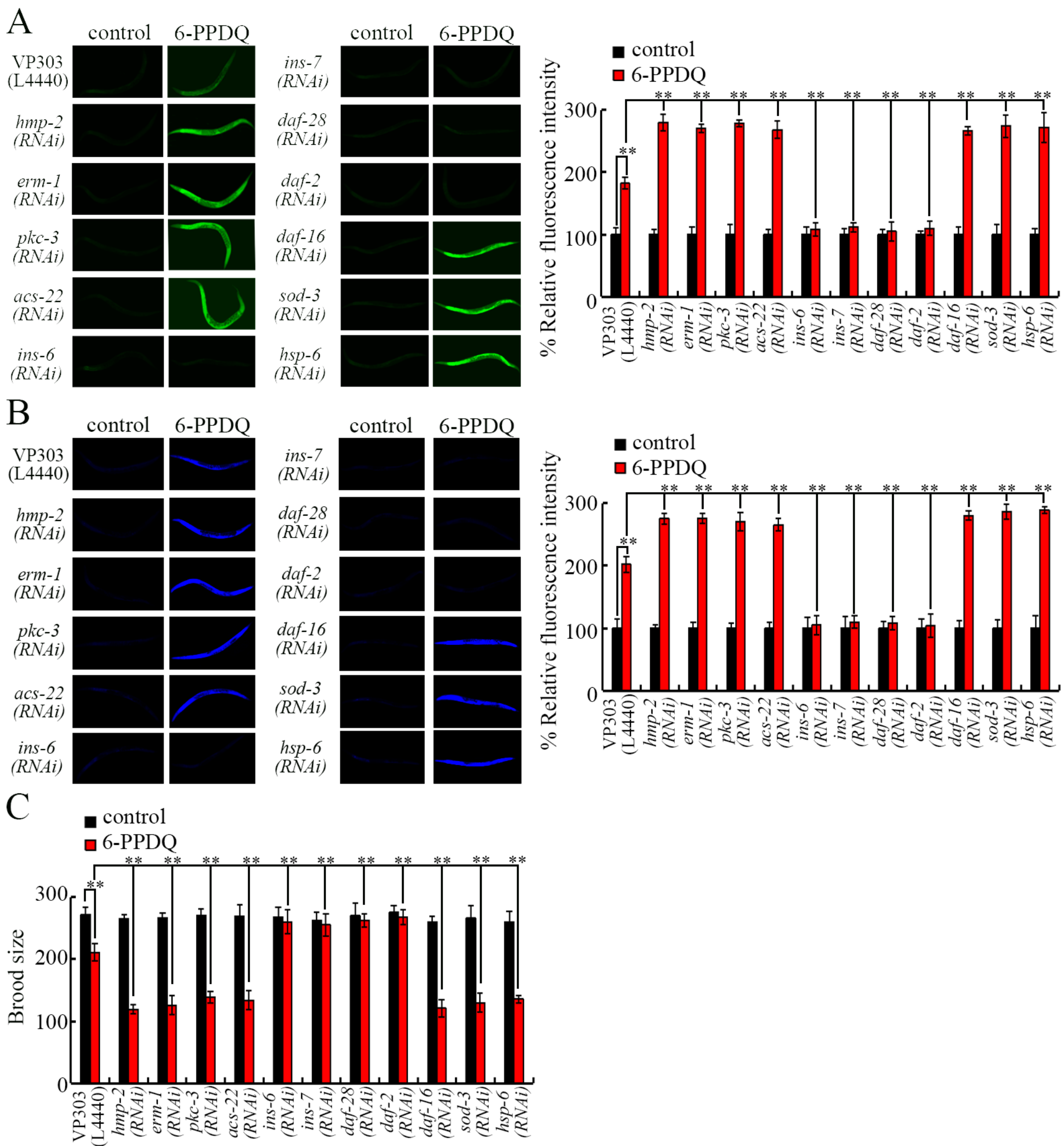


**Figure S3.** Effect of intestinal RNAi of *hmp-2*, *erm-1*, *pkc-3*, *acs-22*, *ins-6*, *ins-7*, *daf-28*, *daf-2*, *daf-16*, *sod-3*, and *hsp-6* on 6-PPDQ toxicity in causing intestinal ROS generation (A), in inducing intestinal lipofuscin accumulation (B), and in reducing brood size (C). Exposure concentration of 6-PPDQ was 10 μg/L. $^{**}P < 0.01$.

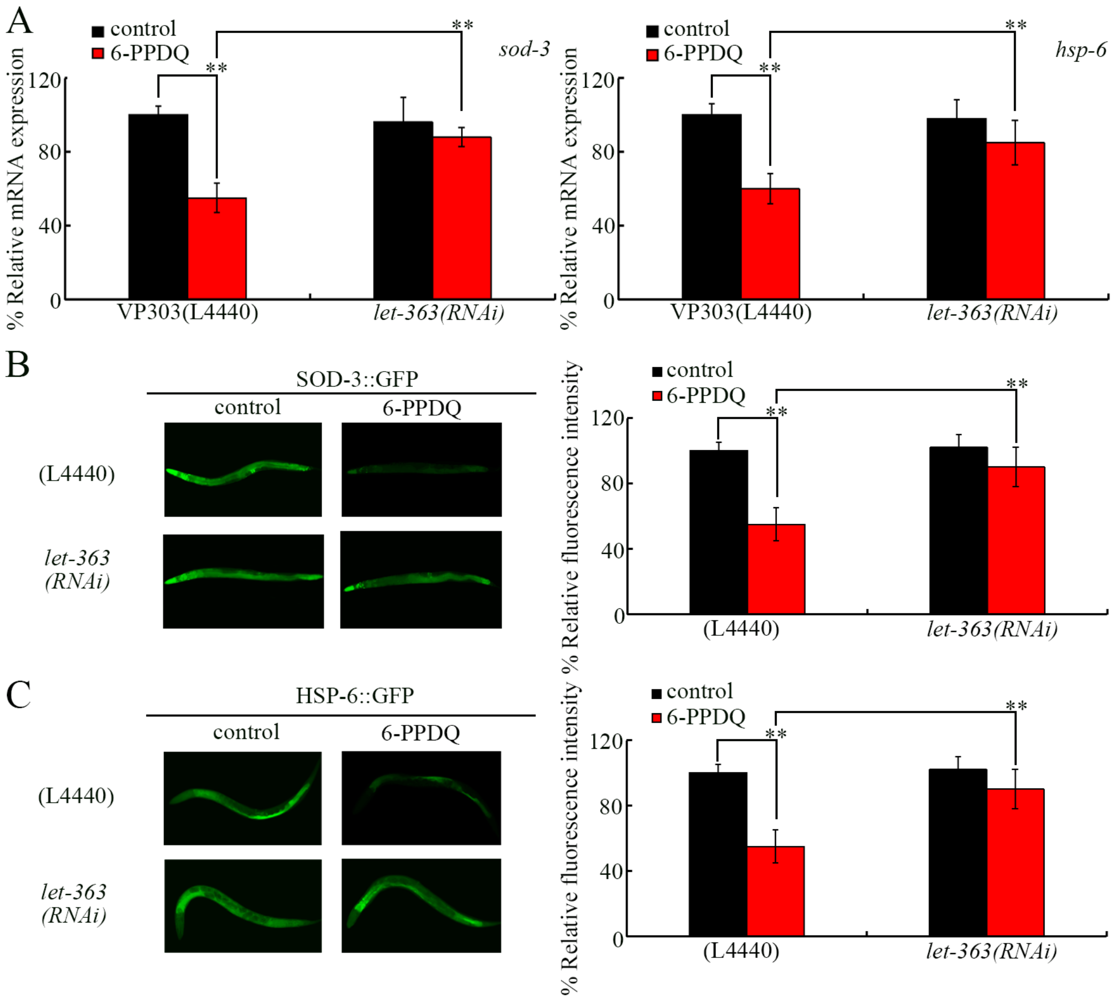


**Figure S4.** Effect of RNAi of *let-363* on expression of SOD-3 and HSP-6 in 6-PPDQ exposed nematodes. (A) Effect of RNAi of *let-363* on expression of *sod-3* and *hsp-6* in 6-PPDQ exposed nematodes. (B) Effect of RNAi of *let-363* on expression of SOD-3::GFP in 6-PPDQ exposed nematodes. (C) Effect of RNAi of *let-363* on expression of HSP-6::GFP in 6-PPDQ exposed nematodes. Exposure concentration of 6-PPDQ was 10 μg/L. $^{**}P < 0.01$.

**Table S1.** Information for *C. elegans* strains

| Strains | Genotype | Description |
|---|---|---|
| N2 | | Wild-type |
| CF1553 | *muIs84 [(pAD76) sod-3p::GFP + rol-6(su1006)]* | Transgenic strain expressing SOD-3::GFP |
| SJ4100 | *zcIs13 [hsp-6p::GFP + lin-15(+)]* | Transgenic strain expressing HSP-6::GFP |

**Table S2.** Primer information for qRT-PCR

| Gene | Forward primer (5'-3') | Reverse primer (5'-3') |
|---|---|---|
| *acl-1* | GCTCCACGAGATTGTCACCA | GGCGTGGCTGTAGAAGAACT |
| *acl-2* | TTCTACTCAAAGCCAGGCCG | AACCCGAAACGGAAAGGACA |
| *acl-3* | CACGATGTCGGGTACAATGC | GCAAATGTTGTGAGCTGCGA |
| *acl-4* | GGTAGACAAGCCGGTATCTGG | GTGGTGGGGAGGTGATCAAG |
| *acl-5* | AAGCTTGTGACACTCCAGCA | GGAACCGTTCAAACGTAAACAA |
| *acl-6* | TGAAGCAGAACAGGCATTCCA | ACTCCAAGCACAGACTCACG |
| *acl-7* | TTATCGCTTTTCCGGTGGCT | TCTGCACAGTTCCCACCATC |
| *acl-8* | AGAGCGAGCAACTCGTTTGA | CTAGTCGCTCTTCCTGCTCG |
| *acl-11* | GCAGGCTTTTCGTGTGATGG | TGGTTGACGGCCACTTTTCT |
| *acl-14* | CCAATGGGCAGCGACCAC | TGTACGTGCCCACATAACGG |
| *act-5* | CAGGGAGTGATGGTCGGTAT | CGGTAAGGAGAACTGGGTGT |
| *hmp-2* | AACCGGCGTTGATCAGTCTT | AGTACAGTGCCGAAGAGCAC |
| *erm-1* | GTTCTTCTTGCCTCTTACGC | TATTACGAATCTCCGACCAT |
| *pkc-3* | GGCTTCCGTGTCAAGGAGAA | AGAGAACCACTTGCAGGAGC |
| *acs-22* | TCATTCGGCTGTGCAAGGAA | TGAGCCGAGAGGGAAATGAG |
| *ins-6* | GGAATCAGCTCACCGACCAA | TGAGACACGGGTGAAACGAG |
| *ins-7* | TTTCTAATCAATACGATGCCACCA | TGAAGTCGTCGGTGCATTCT |
| *daf-28* | GCTCATCGCCATCTTTGC | ACCTGGAACACGGACTGC |
| *daf-2* | GCTTACGCGATGAGCTGTGAT | TCGCTGGCGACTATGTGA |
| *daf-16* | ACATTGCTCGAAGTGCCGAA | CATTGCTGTCGACCCGTTTG |
| *sod-3* | CAATTGCTCTCCAACCAGCG | TTTGCACAGGTGGCGATCTT |
| *hsp-6* | ACAGGCCATGCAGAGACTTC | CTTGAACAGTGGCTTGCACC |
| *let-363* | GAGAACGACCACATCCAA | AAGACCGACGAGTGAACC |
| *tba-1* | TCAACACTGCCATCGCCGCC | TCCAAGCGAGACCAGGCTTCAG |
